# Why PLoS Sponsored a Roundtable of Medical Whistleblowers

**DOI:** 10.1371/journal.pmed.0020208

**Published:** 2005-05-27

**Authors:** The PLoS Medicine Editors

## Abstract

On May 15, 2005, PLoS and the Government Accountability Project co-sponsored a roundtable of some of the most high profile medical whistleblowers of recent times.

On May 15, 2005, the Public Library of Science and the Government Accountability Project, a public interest legal group that advises and supports whistleblowers (www.whistleblower.org), co-sponsored a private meeting near the Capitol Building in Washington, D. C. In the room were four of the most high profile medical whistleblowers of recent times. All four have gone public with information about practices in medicine and medical research that they believe are risking the public's health or safety [1–5]. One of them was David Graham, Associate Director in the United States Food and Drug Administration's (FDA's) Office of Drug Safety, whose research on rofecoxib (Vioxx) pointed to the serious cardiovascular risks of the drug [1]. Graham was speaking in his own capacity and was not representing the FDA. An anonymous fifth whistleblower—a research scientist at a major drug company—participated by phone.

The whistleblowers took turns to share their stories, including their accounts of retaliations they said they had faced from their employers on raising their concerns, which led to lawsuits by at least two of the whistleblowers [2,3]. The picture that emerged from these accounts—a picture of American medicine's inappropriate ties with the pharmaceutical industry—was deeply troubling.

As the investigative medical journalist Jeanne Lenzer reports in her Essay for *PLoS Medicine* [6], the whistleblowers spoke of public regulatory agencies that are putting the interests of drug companies ahead of the safety of patients, and of pharmaceutical companies that allow their marketing departments to knowingly downplay serious side effects when promoting their drugs. And they spoke of the woefully inadequate protection offered to those in the medical community who feel morally compelled to blow the whistle.

It was Lenzer who conceived the idea for the meeting. She believed that important lessons would emerge from having these medical whistleblowers, who come from very different professional backgrounds, together in one room to share their experiences. It took her many months of planning. In particular, she needed to gain the trust of the industry research scientist, so that the scientist could feel sure that anonymity would be preserved. But all of her planning nearly came to nothing. At the last moment the original journal sponsor pulled out on the advice of its lawyers.

*The picture that emerged from these accounts was deeply troubling.*

Lenzer's phone call to PLoS, enquiring whether we might step in, came just ten days before the event was due to happen. We took our own legal advice and then agreed to sponsor the roundtable. PLoS was eager to support this event, and willing to accept any small legal risks, because we believe that the issues raised will be of huge interest to the medical community, to the press, to patients, and to the broader public. Further, the event fits well with our own public service mission of making all scientific and medical research results freely and publicly available, and with our belief that transparency in the conduct and publication of research is important for public trust. And the topic of the roundtable was in line with other articles we have published highlighting the many ways in which medicine has become tightly entangled with industry, to the great detriment of patients [7–9].

The risks to a journal in sponsoring such an event are, of course, much smaller than the risks that the whistleblowers at the roundtable faced in going public with their stories. Studies have shown that whistleblowers in both public service and private industry almost always experience retaliation from their employers, with those employed longer experiencing greater retaliation [10,11]. They risk loss of earnings, intimidation, harassment, victimization, and personal abuse, and they traditionally receive little help from statutory authorities [12,13].

The Washington whistleblowers' stories illustrate these issues. Psychiatrist Stefan Kruszewski described how he was fired from his job at the Pennsylvania Department of Public Welfare (DPW) after alerting his seniors to prescribing practices across the state that he considered to be alarming and dangerous [2]. “I was fired in a demeaning manner,” said Kruszewski, who has sued DPW over his firing. “My two offices were emptied and the contents of these offices were put in the gutter.” David Graham—who testified at a US Senate Finance Committee hearing on rofexocib (Vioxx), the FDA, and Merck [1]—said that there was a conspiracy by senior management at the FDA “to intimidate me ahead of the Senate testimony.” Both of these individuals contend that pharmaceutical industry influence over their employers (a state and a federal regulatory agency, respectively) played a part in the difficulties these individuals faced in getting their concerns heard [1,2].

Lenzer's report will, we hope, spark discussion and debate about how American medicine—clinicians, researchers, regulatory agencies, and medical journals—can disentangle itself from the influence of the pharmaceutical industry. In the past, medical journals and their editors have played an important part in exposing the complex relationships between the pharmaceutical industry and medicine [14–16], including between industry and the medical journals themselves [9]. *PLoS Medicine* will continue to look critically at these relationships. A common theme at the roundtable was that, armed with information, the public too could have an important role in unpicking these ties. “The pharma–FDA complex has to be dismantled,” said Graham, “and the American people have to insist on that, otherwise we're going to have disasters like Vioxx that happen in the future.” Patients, health professionals, and even the industry itself all surely stand to gain from disentanglement.
